# Decreased sensitivity to the anorectic effects of leptin in mice that lack a *Pomc*-specific neural enhancer

**DOI:** 10.1371/journal.pone.0244793

**Published:** 2020-12-31

**Authors:** Elisa S. Na, Daniel D. Lam, Eva Yokosawa, Jessica M. Adams, David P. Olson, Malcolm J. Low

**Affiliations:** 1 Department of Psychology & Philosophy Texas Woman’s University, Denton, Texas, United States of America; 2 Institute of Neurogenomics, Helmholtz Center Munich, German Research Center for Environmental Health, Neuherberg, Germany; 3 Chair of Neurogenetics, Neurological Clinic and Polyclinic, Klinikum rechts der Isar, School of Medicine, Technical University of Munich, Munich, Germany; 4 Department of Molecular & Integrative Physiology, University of Michigan Medical School, Ann Arbor, Michigan, United States of America; 5 Division of Endocrinology, Department of Pediatrics, University of Michigan Medical School, Ann Arbor, Michigan, United States of America; Hospital Infantil Universitario Nino Jesus, SPAIN

## Abstract

Enhancer redundancy has been postulated to provide a buffer for gene expression against genetic and environmental perturbations. While work in Drosophila has identified functionally overlapping enhancers, work in mammalian models has been limited. Recently, we have identified two partially redundant enhancers, nPE1 and nPE2, that drive proopiomelanocortin gene expression in the hypothalamus. Here we demonstrate that deletion of nPE1 produces mild obesity while knockout of nPE2 has no discernible metabolic phenotypes. Additionally, we show that acute leptin administration has significant effects on nPE1 knockout mice, with food intake and body weight change significantly impacted by peripheral leptin treatment. nPE1 knockout mice became less responsive to leptin treatment over time as percent body weight change increased over 2 week exposure to peripheral leptin. Both *Pomc* and *Agrp* mRNA were not differentially affected by chronic leptin treatment however we did see a decrease in *Pomc* and *Agrp* mRNA in both nPE1 and nPE2 knockout calorie restricted mice as compared to calorie restricted PBS-treated WT mice. Collectively, these data suggest dynamic regulation of *Pomc* by nPE1 such that mice with nPE1 knockout become less responsive to the anorectic effects of leptin treatment over time. Our results also support our earlier findings in which nPE2 may only be critical in adult mice that lack nPE1, indicating that these neural enhancers work synergistically to influence metabolism.

## Introduction

The arcuate nucleus of the hypothalamus plays a critical role in integrating neuroendocrine signals regarding nutritional status and serves as an important site for maintenance of body energy homeostasis [1]. The arcuate nucleus contains a heterogeneous population of neurons, including first-order neurons co-expressing proopiomelanocortin (POMC) and cocaine- and amphetamine-regulated transcript (CART) anorexigeic neuropeptides as well as orexigenic co-expressing neuropeptides neuropeptide Y (NPY) and agouti-related peptide (AgRP) [2]. These neuropeptides work in an opposing fashion to maintain energy balance at optimal levels. POMC neurons, in particular, regulate a variety of physiological processes such as glucose homeostasis, cardiovascular function, lipid metabolism and appetite [3–5] and as such have a prominent role in the regulation of food intake. Loss-of-function *Pomc* mutations results in marked obesity and metabolic dysfunction, demonstrating that *Pomc* has a critical role in metabolism [6–11]. Recently, we identified two phylogenetically conserved mammalian enhancers (nPE1 and nPE2) that drive *Pomc* gene expression [12–14]. Simultaneous deletion of nPE1 and nPE2 leads to near complete abolishment of arcuate *Pomc* expression and compromised metabolic function, increased adiposity, hyperphagia and reduced expression of the melanocortins, α-MSH and β-endorphin, as well as morbid obesity [13]. Interestingly, single knockout (KO) of nPE1 produces an overweight phenotype in mice but knockout of nPE2 has no discernible impact on metabolism or feeding behavior. These data indicate an additive effect of nPE1 and nPE2 whereby knockout of both enhancers leads to maladaptive behaviors such as hyperphagia and the subsequent development of morbid obesity while single mutations of these enhancers results in mild effects, as observed in nPE1 KO mice, to no overt phenotypic effects, as is the case in nPE2 KO mice. Presumably, complete redundancy is apparent if simultaneous inactivation of both enhancers produces deleterious consequences. These experiments, however, revealed partial redundancy as inactivation of each alone had mild phenotypic effects. Past work in Drosophila has demonstrated that redundant enhancers perform a mitigating role against environmental and genetic disturbances [15,16], thereby ensuring the “canalization” of developmental processes [16,17]. Regulatory redundancy has been postulated to buffer against adverse environmental or genetic disturbances and enhances phenotypic robustness so as to confer an adaptive advantage [18–20]. The partial redundancy then of nPE1 and nPE2 suggests that these enhancers have overlapping functions and that dysfunction of one enhancer can be counteracted by the intact function of its paralogue [13].

Leptin is a hormone produced by adipocytes that suppresses food intake by binding to its receptor LepR in a variety of brain regions including POMC and AgRP neurons [21]. Humans and mice lacking leptin have hyperphagic obesity, hyperinsulinemia, and decreased energy expenditure [22,23] demonstrating the prominent role that leptin plays in energy homeostasis. Past studies have demonstrated that leptin activates POMC neurons to produce its anorectic effects [24]. We have shown previously that reactivation of *Pomc* expression to LepR^+^-specific neurons rescues food intake, body weight and locomotor activity in previously *Pomc* deficient mice [25]. These data show that POMC neurons are essential for leptin action on feeding behavior and that LepR-expressing POMC neurons are necessary for the establishment of normal body energy homeostasis.

Here, we sought to elucidate the effect of these nPEs on behavioral responses to leptin to determine if knockout of either one of these neural enhancers produces effects on body weight phenotype and feeding behavior in a leptin-dependent fashion. We also sought to characterize the contribution of nPEs to leptin-dependent *Pomc* expression. We demonstrate that nPE1 KO mice are more responsive to acute leptin treatment in terms of food intake and body weight. However, with continuous leptin infusion over 2 weeks, this effect was reversed and nPE1 KO mice became leptin insensitive. In contrast, we did not observe any differential leptin responses in nPE2 KO mice. We postulate that the leptin insensitivity in our nPE1 KO mice, chronically treated with leptin, may be a function of the integration of distinct cytokine signaling pathways in POMC neurons by nPE1 whereby nPE1 may dynamically regulate downstream mechanisms associated with leptin signaling.

## Methods

### Animals

Adult male mice were housed in ventilated cages under controlled temperature and humidity conditions. Mice were maintained on a 12 hr light/dark cycle (lights on from 06:00 to 18:00) with laboratory chow containing 28.0% kcal protein, 12.1% kcal fat, and 59.8% kcal carbohydrate and tap water available *ad libitum* unless otherwise noted. nPE1 KO and nPE2 KO mice were used and wildtype mice were siblings of the nPE1 KOs. All mice were backcrossed to C57BL/6J for > 10 generations [13,26]. All experiments were approved by the University of Michigan Institutional Animal Care and Use Committee (IACUC) and followed the Public Health Service guidelines for the humane care and use of experimental animals.

### Calorie restriction

Mice were calorie restricted until body weights reached 20% below baseline levels. Mice were maintained at this body weight for the duration of the study.

### Leptin administration

Mice were injected with 5 mg/kg leptin (MedImmune), i.p. or PBS 3 times over a 24 hr period. Body weight (BW) and food intake were determined immediately before and 2 hrs after the final leptin/PBS injection. To measure food intake, mice were given *ad libitum* access to food over the course of leptin treatment. Mice were randomly assigned to one of 6 groups: 1) WT PBS (n = 12); 2) WT leptin (n = 11); 3) nPE1 KO PBS (n = 10); 4) nPE1 KO leptin (n = 9); 5) nPE2 KO PBS (n = 9); 6) nPE2 KO leptin (n = 10).

For chronic leptin administration, PBS or leptin (5 mg/kg/day) were infused using 14 day osmotic minipumps (Alzet, Durect Corporation) and were implanted in between the scapulae of WT, nPE1 KO, and nPE2 KO mice under isoflurane anesthesia (Henry Schein, induction rate 3%; maintenance rate 1%). Mice were randomly assigned to one of 3 groups: 1) WT calorie restriction (CR) (n = 20); 2) nPE1 KO CR (n = 17); 3) nPE2 KO CR (n = 16).

### *In situ* hybridization

For the acute leptin experiment, adult WT, nPE1 KO, and nPE2 KO mice were given 3 injections of PBS or leptin, spaced 8 hours apart, beginning at 4 pm. Eighteen to twenty hours after the first leptin/PBS injection, mice were sacrificed. Brains were extracted and rapidly frozen in isopentane on dry ice. Frozen brains were cut on a cryostat at 16 μm at -19-20°C and mounted onto DEPC gelatin-coated slides. Slides were fixed in 10% buffered formalin for 1 hr after which time they were washed with DEPC-treated PBS. Brain sections were then acetylated for 10 min with 0.1 M triethanolamine and acetic anhydride before washing with 2x SSC. Slides were dehydrated in ethanol washes (50%, 70%, 95%, and 100%) for 4 min each at RT and then air dried for 30–60 min. *Pomc* [27] and *Agrp* [28] radiolabeled probes diluted with hybridization solution (Denhardt’s solution, formamide, 50% dextran sulfate solution, 1 M Tris pH 8, and 0.5 M EDTA pH 8) were heated at 65°C for 5 min before application to slides. Slides were coverslipped and sealed with DPX and hybridized overnight at 60°C. The following day coverslips were removed by soaking with 4x SSC for 1 hr. Slides were exposed to an RNase digestion step (500 μL RNase A, 10 mg/mL, 50 mL 5 M NaCl, 5 mL 1M Tris pH 8, 1000 μL 0.5M EDTA pH 8, 443.5 mL mQ H_2_O) for 25 min at 37°C before stringency washes in descending concentrations of SSC (2x, 1x, 0.5x, and 0.1x) and DTT. Slides were then dehydrated in an ascending series of ethanol concentrations (50%, 70%, 95%, and 100%) for 3 min each at RT before air drying for 30–60 min. Brain sections were exposed to a Phosphorimager screen before scanning on a Phosphorimager. *Pomc* and *Agrp* mRNA densitometry values were quantified using TotalImage Quant. The three highest densitometry values were used for each brain and averaged. Values were normalized to the control group and are represented as percent of control values.

### qRT-PCR

The mediobasal hypothalamus was dissected as follows. First, using a brain matrix and razor blades, a coronal section encompassing the entire hypothalamus was prepared. The section was then oriented coronally and trimmed vertically at the cortical-hypothalamic ventral invagination and horizontally at the fornix. RNA was extracted with RNeasy columns (Qiagen) and reverse transcribed with random primers using GoScript Reverse Transcription system (Promega). A FAM-labeled *Pomc* probe (Mm00435874_m1; Life Technologies) and a VIC-labeled 18S rRNA probe (Mm03928990_g1; Life Technologies) were used in multiplex and quantitation was performed by the 2^−ΔΔCT^method [13]. Mice were randomly assigned to one of 6 groups: 1) WT PBS (n = 6); 2) WT leptin (n = 6); 3) nPE1 KO PBS (n = 6); 4) nPE1 KO leptin (n = 6); 5) nPE2 KO PBS (n = 6); 6) nPE2 KO leptin (n = 6).

### Statistics

One way ANOVAs were used to analyze acute leptin treatment data as well as qPCR data. A two-way ANOVA was used to analyze the *in situ* hybridization data. A 2x3 repeated measures ANOVA was used to assess the effects of long-term leptin treatment on body weight lost and percent change in food intake. Fisher’s LSD post-hoc tests were used when appropriate. Cohen’s *d* was used to calculate magnitude of group differences as appropriate [29]. Statistical outliers were identified using a Grubbs’ outlier test (GraphPad Prism). Statistics were analyzed using SPSS software (version 25). *P* values less than or equal to 0.05 were considered statistically significant.

## Results

### Acute leptin treatment

**Fig 1:**
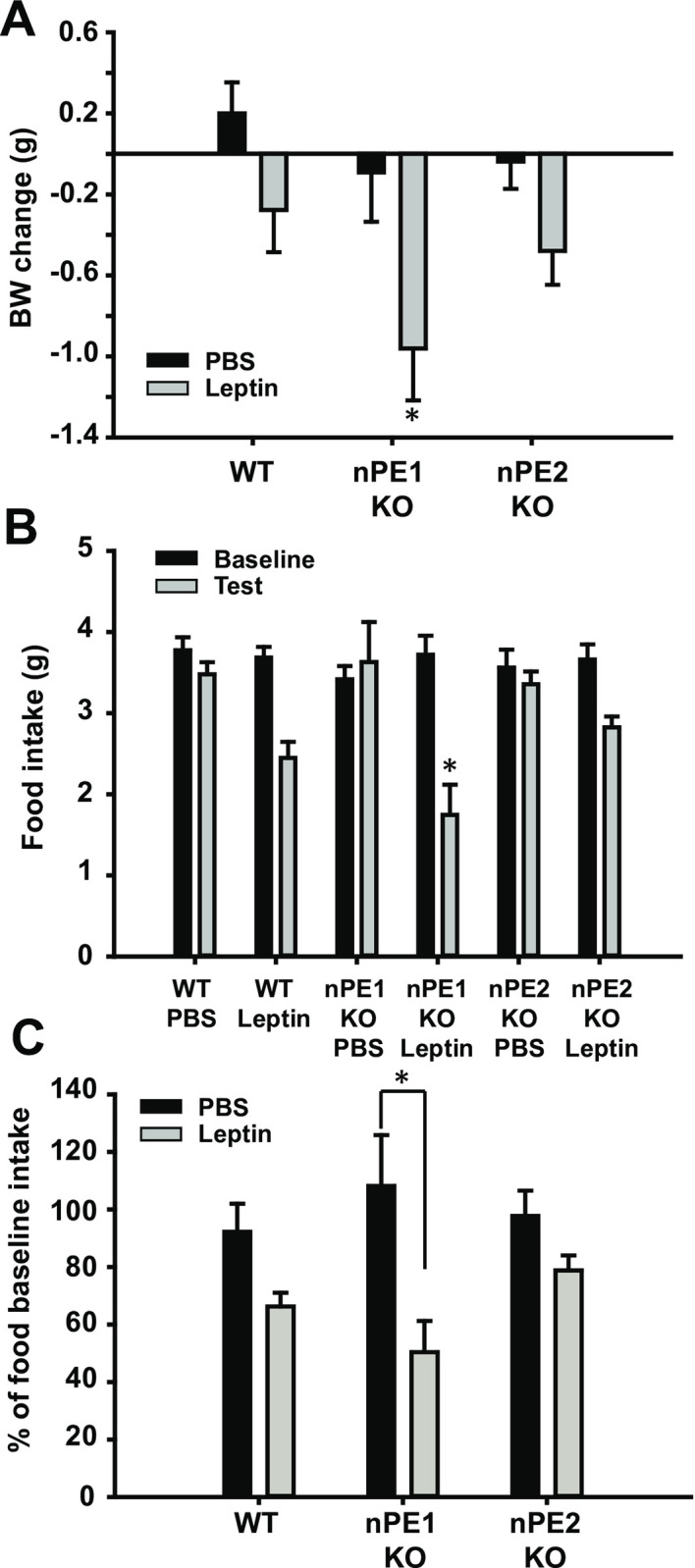
Effects of acute leptin treatment on food intake and body weight. A. Acute leptin treatment decreases body weight (BW) in wildtype (WT), nPE1 KO, and nPE2 KO groups, with significant differences between the nPE1 KO group and all other groups with the exception of the nPE2 KO leptin group. B. Food intake is significantly attenuated in nPE1 KO mice after acute peripheral leptin treatment as compared to all other groups with the exception of the WT leptin-treated mice. C. nPE1 KO mice treated with leptin show significantly attenuated percent food intake relative to their baseline intake compared to nPE1 KO PBS-treated group, indicating that nPE1 KO mice are sensitive to the anorectic effects of peripheral leptin treatment. * *p*<0.05.

In order to assess responsiveness to exogenous leptin treatment, mice were given either intraperitoneal leptin or PBS injections. nPE1 KO mice were more sensitive to the acute anorectic effects of leptin as demonstrated by significant reductions in food intake and body weight (Fig 1). Specifically, a one-way ANOVA revealed significant differences between groups in food intake and in body weight change after acute PBS or leptin treatment, *F*(_5,54_) = 8.29, *p*<0.01 and *F*(_5, 54_) = 4.31, *p*<0.01, respectively. Fisher’s LSD post-hoc analyses show group differences in body weight change, with nPE1 KO leptin-treated mice losing significantly more weight compared to all other groups except the nPE2 KO leptin-treated mice (*p*<0.05; Fig 1A). Based on a Grubbs’ outlier test, we detected 1 significant outlier in the WT PBS, nPE1 KO PBS, and nPE1 KO leptin-treated groups and those 3 mice were excluded from all statistical analyses.

We also observed group differences in acute food intake after leptin/PBS treatment between nPE1 KO leptin-treated mice compared to all other groups with the exception of WT leptin-treated mice (*p* = 0.054), indicating that food intake in nPE1 KO mice is significantly attenuated by leptin treatment (Fig 1B). As expected, we observed changes in food intake relative to baseline values as assessed by a one-way ANOVA, (*F*(_5,54_) = 3.92, *p*<0.01) with group differences between nPE1 KO leptin-treated group and nPE1 KO vehicle-treated mice (Fig 1C).

**Fig 2:**
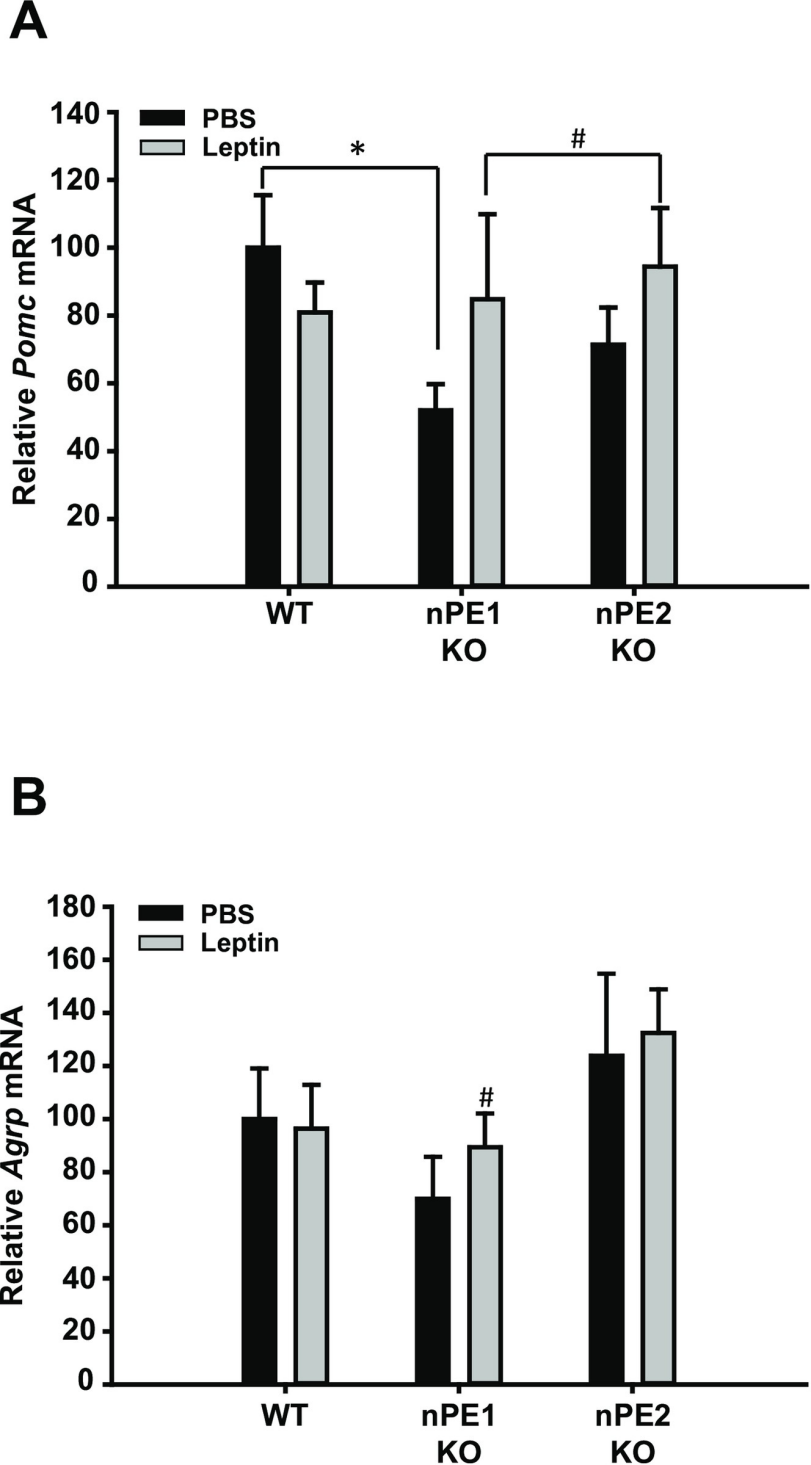
Effects of leptin treatment on expression of *Pomc* and *Agrp* mRNA in the hypothalamus in *ad libitum* fed mice as assessed by in situ hybridization. A. A two-way and one-way ANOVA did not reveal significant group differences in *Pomc* mRNA expression in the arcuate nucleus of mice given *ad libitum* access to food. A t-test comparing the WT and nPE1 KO PBS groups replicate earlier findings that *Pomc* mRNA is significantly decreased in nPE1 KO mice as compared to WT littermate controls. In order to determine group differences, a Cohen’s *d* test was used. A substantial effect size was found between nPE1 KO vehicle and nPE1 KO leptin-treated groups, *d* = 0.67. B. *Agrp* mRNA is not differentially affected by acute leptin treatment in the arcuate nucleus between *ad libitum* fed mice but based on a Cohen’s *d* analysis, there is a substantial group difference between nPE1 KO vehicle and nPE1 KO leptin-treated mice, *d* = 0.71. * *p*<0.05 between WT PBS and nPE1 KO PBS groups. # *d*≥0.5 between nPE1 KO vehicle and nPE1 KO leptin-treated groups.

To determine if the effect of leptin is dependent on the animal’s energy status, we also measured *Pomc* and *Agrp* mRNA expression following acute leptin administration in *ad libitum* fed animals. We did not observe any significant effects in *Pomc* or *Agrp* mRNA expression as a function of leptin treatment as assessed by *in situ* hybridization in *ad libitum* fed mice. A t-test comparing *Pomc* expression between the WT vehicle group and the nPE1 KO vehicle group confirmed our previous findings indicating that arcuate *Pomc* mRNA is significantly decreased in nPE1 KO mice [13]. In order to determine the magnitude of difference between nPE1 KO groups, we used Cohen’s *d* and found that there was a substantial effect size in *Agrp* and *Pomc* mRNA expression between the nPE1 KO vehicle-treated and nPE1 KO leptin-treated groups given *ad libitum* access to food (Fig 2), *d* = 0.71 and *d* = 0.67, respectively.

**Fig 3:**
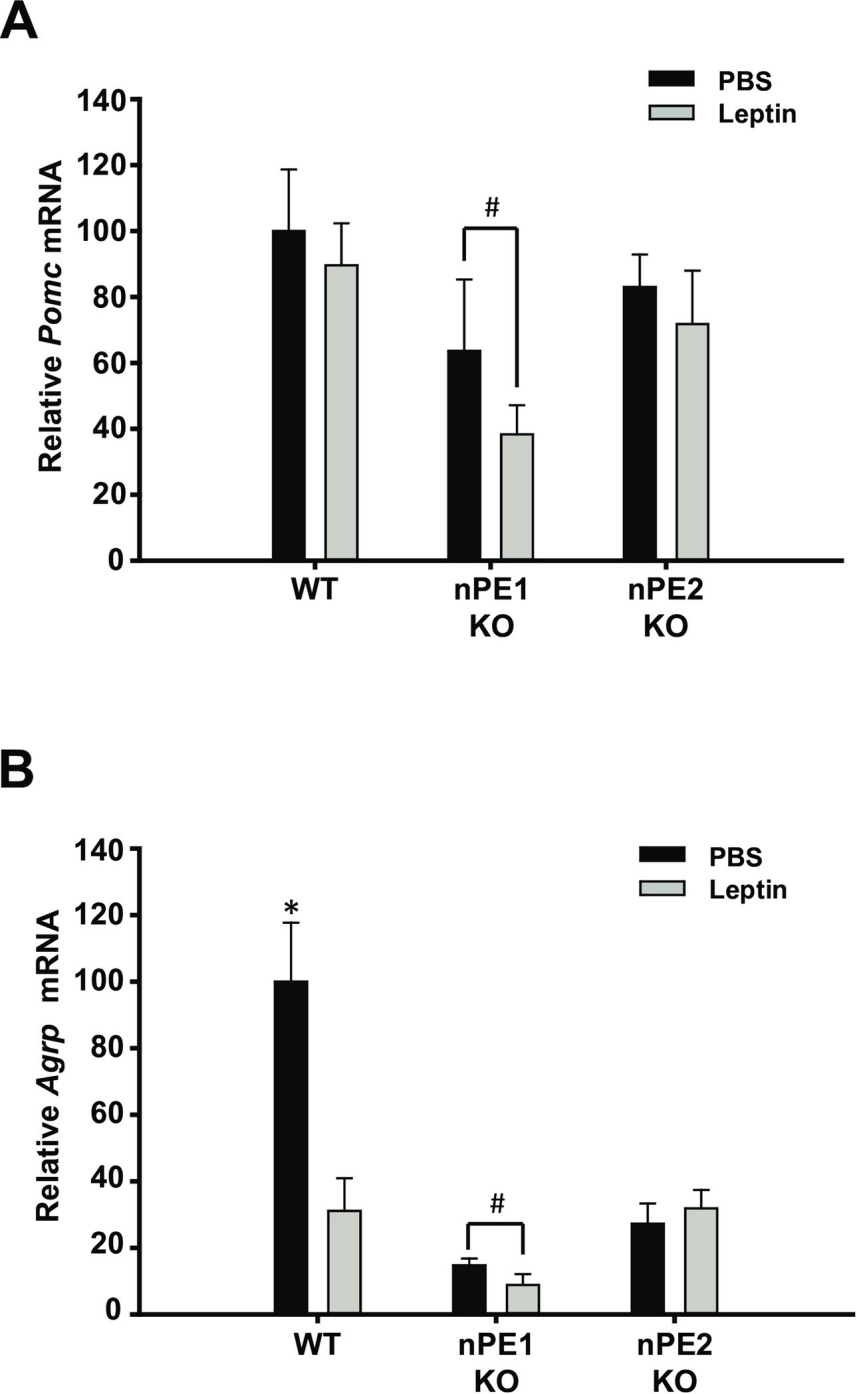
Acute leptin treatment in chronically food restricted mice does not significantly affect hypothalamic *Pomc* or *Agrp* mRNA between genotypes as demonstrated by qPCR. A. *Pomc* mRNA is not altered between WT and KO groups when comparing food restricted mice. Based on a Cohen’s *d* analysis, there was a substantial group difference between the two nPE1 KO groups, *d* = 0.53. B. *Agrp* mRNA is not substantially affected by leptin administration but there is a significant decrease in *Agrp* mRNA in all groups compared to the WT PBS-treated group. There was also substantial group differences between nPE1 KO groups as revealed by a Cohen’s *d* test, *d* = 0.733. * *p*<0.05 indicates significance. #d≥0.5 between nPE1 KO PBS and nPE1 KO leptin-treated mice.

To determine if the effect of leptin is dependent on the animal’s energy status, we also measured *Pomc* and *Agrp* mRNA expression following leptin administration in calorie-restricted animals. A one-way ANOVA did not reveal significant between group differences in *Pomc* mRNA expression (Fig 3A) however, we show that *Agrp* mRNA is significantly decreased in all groups as compared to the WT PBS-treated group as determined by a one-way ANOVA, *F*(_5,30_) = 13.2, *p*<0.01(Fig 3B). Using a Fisher’s LSD, we show that all groups have decreased *Agrp* mRNA expression as compared to the WT PBS-treated group but because group differences could not be determined using Fisher’s LSD, we used Cohen’s *d* to examine the magnitude of differences between nPE1 KO groups. Based on our Cohen’s *d* analysis, we found a substantial effect size in *Agrp* and *Pomc* mRNA expression between the nPE1 KO vehicle and nPE1 KO leptin-treated groups, *d* = 0.733 and *d* = 0.53, respectively.

### Chronic leptin treatment

**Fig 4:**
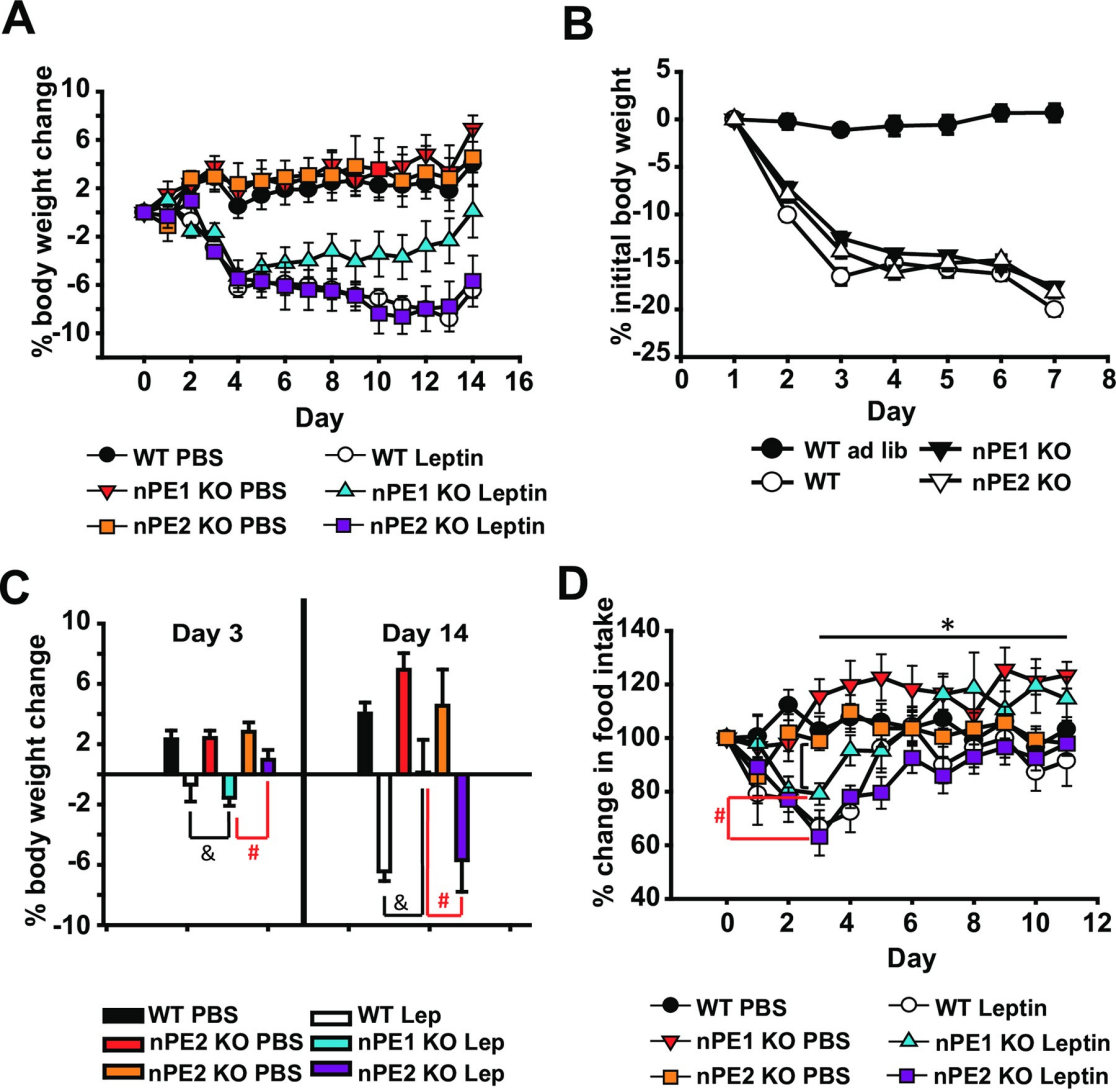
nPE1 KO mice become less sensitive to the anorectic effects of long-term exposure to leptin treatment. A. WT and nPE1 KO mice lose comparable amounts of weight after the first week of leptin treatment however over time nPE1 KO mice become less sensitive to peripheral leptin by day 14 of treatment with nPE1 KO leptin-treated mice having a significant increase in body weight change compared to WT PBS, nPE1 KO PBS, nPE2 KO PBS, and nPE2 KO leptin groups. B. There are no significant differences in percent initial body weight change over the first week of food restriction indicating that all genotypes lost body weight at similar rates and that body weight loss is not necessarily a function of genotype. C. Peripheral leptin exposure significantly decreased percent body weight in *ad libitum* fed nPE1 KO mice as well as the WT control group on day 3 of leptin treatment as compared to nPE1 KO and WT vehicle groups, respectively. There was also a significant difference between the nPE1 KO leptin-treated group and the nPE2 KO leptin-treated group, in that the nPE1 KO leptin-treated group lost more percent body weight compared to the nPE2 KO leptin-treated group. However over time, nPE1 KO mice become less responsive to leptin such that percent body weight increases by the 14^th^ day of leptin exposure as compared to the WT and nPE2 KO leptin-treated groups. D. There were significant differences in food intake between the third and last day of leptin treatment in our nPE1 KO mice, indicating that these mice significantly increased percent food intake over time despite leptin treatment. There are also significant differences between nPE1 KO leptin-treated mice and nPE2 KO leptin-treated mice in percent food intake, with nPE1 KO leptin-treated mice eating significantly less than nPE2 KO leptin-treated mice on day 3 of leptin treatment. * *p*<0.05 indicates significance between third and last day of leptin for nPE1 KO mice (Fig 4D). #*p*<0.05 indicates significance between nPE1 KO leptin and nPE2 KO leptin-treated groups. & *p*<0.05 indicates significance between WT leptin and nPE1 KO leptin-treated groups.

Given that nPE1 KO mice were behaviorally more sensitive to the acute anorectic effects of leptin treatment, we sought to determine if nPE1 KO mice were sensitive to the chronic effects of leptin treatment. In this experiment, mice were implanted with osmotic minipumps and body weight as well as food intake were measured over the course of approximately 2 weeks. We found that a 2x3 repeated measures ANOVA did not show a significant interaction effect between time, drug (PBS, leptin) and genotype (WT, nPE1 KO and nPE2 KO groups) (Fig 4A). We observed significant interaction effects between time and drug (*F*(_14,378_) = 28.9, *p*<0.01) as well as time and genotype (*F*(_28,378_) = 8.6, *p*<0.01).

Our data demonstrate no appreciable differences in percentage body weight lost after food restriction, suggesting that all mice lose weight in a similar fashion and that body weight loss is not a function of genotype (Fig 4B).

With respect to group differences in initial percentage body weight lost after the third day of vehicle/leptin treatment as well as the last day of vehicle/leptin treatment, a one-way ANOVA revealed significant differences between groups (*F*(_5,32_) = 5.88, *p*<0.01 for day 3 percent body weight lost and *F*(_5,32_) = 12.02, *p*<0.01 for day 14 percent body weight lost; Fig 4C). Additional post-hoc analyses demonstrated significant differences between WT and nPE1 KO leptin-treated groups compared to vehicle-treated groups (*p*<0.05) after the third day of leptin treatment indicating WT and nPE1 KO mice were responsive to the anorectic effects of leptin. Interestingly, nPE1 KO leptin-treated mice appeared to be more acutely sensitive to its anorectic effects as compared to nPE2 KO leptin-treated mice as there were significant differences in percent body weight change between these two groups after 3 days of leptin treatment (Fig 4C). Additionally, we show that nPE1 KO leptin-treated mice appear to be less responsive to leptin treatment by day 14 as compared to both nPE2 KO leptin-treated and WT leptin-treated mice. Post-hoc analyses reveal significant differences between nPE1 KO leptin-treated mice versus nPE2 KO leptin-treated and WT leptin-treated mice (*p*<0.05). There are no differences between WT leptin-treated and nPE2 KO leptin-treated mice (Fig 4C).

A repeated measures ANOVA revealed significant interaction effects between time and group for percent change in food intake, *F*(_55, 473_) = 2.566, *p*<0.001 (Fig 4D). Using a paired t-test, we found significant differences in food intake between the third and last day of leptin treatment in our nPE1 KO mice, indicating that these mice significantly increased percent food intake over time despite leptin treatment (*p* = 0.009). We also show significant differences using an independent t-test between nPE1 KO leptin-treated mice and nPE2 KO leptin-treated mice in percent food intake, with nPE1 KO leptin-treated mice eating significantly less than nPE2 KO leptin-treated mice on day 3 of leptin treatment, (*p* = 0.002, Fig 4D).

## Discussion

In this study, we sought to determine the phenotypic effects of nPE1 or nPE2 inactivation on the anorectic and physiological responses to exogenous leptin treatment. We show that single knockout of nPE1 and nPE2 has dissociable effects on food intake and body weight. Specifically, we demonstrate that acute treatment with peripheral leptin significantly suppresses food intake in nPE1 KO mice as compared toWTand nPE2 KO mice (Fig 1B). These data however could not account for the mild obesity in our nPE1 KO mice. We then decided to examine the effects of long-term leptin treatment to see if this mild obesity in nPE1 KO mice was the result of leptin insensitivity. Consistent with our hypothesis, we show that nPE1 KO mice become less sensitive to the anorectic effects of leptin treatment over time while nPE2 KO mice remain relatively consistent in their responses to leptin treatment; nPE1 KO leptin-treated mice increased food intake, comparable to vehicle-treated nPE1 KO mice, and recovered body weight after 2 weeks of leptin treatment (Fig 4), demonstrating that nPE1 KO mice become leptin insensitive over time. With regard to body weight change, nPE2 KO mice are not statistically different from WT leptin-treated mice when given chronic leptin treatment (Fig 4A), suggesting that nPE2 KO mice maintain an intact leptin response. Surprisingly, we did not see corresponding changes in *Pomc* and *Agrp* mRNA as a function of leptin treatment through *in situ* hybridization or qPCR. Our past data have demonstrated that adult nPE1 deficient mice have significantly decreased levels of α-MSH in the hypothalamus as compared to WT and nPE2 KO mice [13]. Thus, the sensitized behavioral response to acute leptin treatment in our nPE1 KO mice may be due to MSH-independent mechanisms as the mice used in the current experiment presumably had decreased α-MSH levels during the time of testing. We replicated our previous findings indicating an overall decrease in *Pomc* mRNA in our nPE1 KO mice [25,30] as well as a slight decrease in *Pomc* mRNA in our nPE2 KO mice as compared to WT littermates (Figs 2A and 3A).

We propose several possible mechanisms for this observation which are worthy of future study. The first possibility is that nPE1 integrates both suppressive and stimulatory cytokine signals over different timescales. Leptin receptor signaling is subject to acute feedback inhibition by suppressor of cytokine signaling 3 (SOCS3) [31]. Exogenous leptin stimulates SOCS3 expression within hours [32], while impairment of SOCS3 results in increased STAT3 phosphorylation, increased stimulatory effect of leptin treatment on *Pomc* mRNA, and increased acute leptin sensitivity [32]. We postulate that nPE1 may mediate SOCS3 signaling at the *Pomc* locus. Thus, disruption of nPE1 would lead to blunted SOCS3 signaling and may account for the initial increased leptin sensitivity in our nPE1 KO mice. The eventual resistance to chronic leptin treatment that we observe in nPE1 KO mice may then develop through compensatory mechanisms such as upregulation of SOCS3 expression. *Socs3* mRNA levels in the arcuate are increased after prolonged high fat diet exposure [33], while *Socs3* deficiency [32,34] or specific deletion of *Socs3* in POMC neurons [35] leads to enhanced leptin sensitivity and resistance to diet induced obesity. Conversely, arcuate-specific [36] or POMC neuron-specific [37] overexpression of Socs3 leads to obesity. Taken together, these results suggest that SOCS3 in POMC neurons exerts an important influence over leptin’s anorexigenic effects. An alternative potential explanation for our results is that nPE1 KO mice may develop leptin insensitivity independent of SOCS3 signaling, for example via reduced expression of the leptin receptor, LepRb.

Previous studies have shown that food deprivation or food restriction leads to an increase in hypothalamic *Agrp* mRNA expression with a concomitant decrease in *Pomc* mRNA expression [38–41]. While we do not observe a significant effect of leptin on *Pomc* mRNA expression in either of the food restricted nPE1 KO or nPE2 KO groups compared to vehicle-treated KO groups we believe this is due to the interaction between these two enhancers. Our previously published data show a synergistic effect between nPE1 and nPE2 enhancers such that the deletion of both produces a morbidly obese mouse [13]; when both neural enhancers are deleted, there is a precipitous drop in *Pomc* expression, to approximately 10% of WT levels that results in decreased energy expenditure, hyperphagia, and early-onset obesity. Given that we see a substantial difference in *Pomc* mRNA levels between the nPE1 KO vehicle and nPE1 KO leptin-treated groups with elevated levels of *Pomc* mRNA in the leptin-treated nPE1 KOs, then it is plausible that using the double KO mice (nPE1 and nPE2) might produce a similar trend with *Pomc* mRNA expression. Using a different transgenic mouse line in which hypothalamic POMC-deficiency is nearly undetectable, we show no effect of acute leptin injections on body weight or 24 hour food intake [6] indicating that this line of POMC-deficient mice are leptin insensitive. These hypothalamic POMC-deficient mice have an insertion of a neomycin cassette within the POMC enhancer locus and thus are different in that regard to the double knockout (nPE1 and nPE2) mice in which enhancer deletions were done without insertion of a neomycin cassette. These data suggest that double knockout of nPE1 and nPE2 would likewise render these mice immune to the anorectic effects of leptin thereby accounting for the development of morbid obesity in double knockout mice. Future experiments could assess if inactivation of POMC enhancers in general promotes obese phenotypes by producing leptin insensitive mice.

Interestingly, we demonstrate that *Agrp* mRNA levels are significantly attenuated in both nPE1 KO and nPE2 KO groups as compared to WT mice. These transgenic mice have been calorie restricted and presumably this would upregulate *Agrp* mRNA levels, as was observed in the WT PBS group (Fig 3B) and previously published data [42–46]. However, given that we see a substantial decrease in hypothalamic *Agrp* mRNA expression in nPE1 KO and nPE2 KO mice as compared to the PBS WT control group then perhaps loss-of-function mutations in nPE1 and nPE2 lead to maladaptive alterations in this orexigenic population of neurons. According to previously published reports, while POMC and AgRP peptides are expressed in two mutually exclusive cell types in adult animals, during embryonic development there is evidence that a subset of POMC and NPY/AgRP neurons are co-expressed [47], giving credence to the idea that these peptidergic populations share the same developmental origins [48]. Our current results indicate that aberrations in nPE1 and nPE2 function might impact *Agrp* early in development given the shared cellular origins of *Pomc* and *Agrp* [47]. The seemingly paradoxical increase in food intake given these reduced levels of *Agrp* mRNA in our nPE1 KO mice (Fig 4D) might be accounted for by dysregulation of LepR or by other compensatory mechanisms such as upregulation of ghrelin signaling through its receptor, GHS-R1a. It is clear from the current data that knocking out nPE1 and nPE2 enhancers can have downstream effects beyond disruptions in POMC neuronal function, the effects of which have yet to be determined.

In conclusion, we replicate our past findings that individual deletion of nPE1 or nPE2 has dissociable effects on metabolic phenotypes such that nPE1 mutations produce an overweight mouse and decreased expression of hypothalamic *Pomc* mRNA while nPE2 mutations produce slight decrements in *Pomc* mRNA expression with no overt consequences on metabolism or feeding behavior. We also show that nPE1 KO are acutely sensitive to the anorectic effects of leptin, which are evidenced by decreased food intake and subsequent body weight loss, but that over time they become desensitized to chronic leptin treatment as body weight and food intake eventually return to baseline levels. Interestingly, *Agrp* mRNA is significantly decreased in both nPE1 KO and nPE2 KO lines in calorie restricted mice, suggesting that disruptions in nPE1 or nPE2 function may also affect *Agrp* expression as a consequence of the common lineage of POMC and AGRP neurons early in embryonic development [47]. Altogether, our data give insight into the complex interactions between leptin, POMC and AgRP and suggest that the developmental processes involved in orchestrating metabolic phenotypes are sufficiently robust to overcome disruptions in nPEs to ensure adequate nutrient intake. Future research could explore the downstream functional consequences of nPE1 and nPE2 mutations on AgRP neuronal function to determine if such manipulations disrupt signaling between POMC and AgRP neurons.

## Supporting information

S1 Data (ZIP)
